# Progression of echocardiographic parameters and prognosis in transthyretin cardiac amyloidosis

**DOI:** 10.1002/ejhf.2606

**Published:** 2022-07-27

**Authors:** Liza Chacko, Nina Karia, Lucia Venneri, Francesco Bandera, Beatrice Dal Passo, Lodovico Buonamici, Jonathan Lazari, Adam Ioannou, Aldostefano Porcari, Rishi Patel, Yousuf Razvi, James Brown, Daniel Knight, Ana Martinez‐Naharro, Carol Whelan, Candida C. Quarta, Charlotte Manisty, James Moon, Dorota Rowczenio, Janet A. Gilbertson, Helen Lachmann, Ashutosh Wechelakar, Aviva Petrie, William E. Moody, Richard P. Steeds, Luciano Potena, Mattia Riefolo, Ornella Leone, Claudio Rapezzi, Philip N. Hawkins, Julian D. Gillmore, Marianna Fontana

**Affiliations:** ^1^ National Amyloidosis Centre, Division of Medicine University College London, Royal Free Campus London UK; ^2^ Heart Failure Unit Cardiology University Department IRCCS Policlinico San Donato Milan Italy; ^3^ Department for Biomedical Sciences for Health University of Milano Milan Italy; ^4^ Center for Diagnosis and Treatment of Cardiomyopathies, Cardiovascular Department Azienda Sanitaria Universitaria Giuliano Isontina (ASUGI), University of Trieste Trieste Italy; ^5^ Barts Heart Centre, The Cardiovascular Magnetic Resonance Imaging Unit, and the Inherited Cardiovascular Diseases Unit St Bartholomew's Hospital London UK; ^6^ Eastman Dental Institute, University College London London UK; ^7^ Department of Cardiology, Queen Elizabeth Hospital Birmingham University Hospitals Birmingham NHS Foundation Trust and University of Birmingham Birmingham UK; ^8^ Division of Cardiology IRCCS Azienda Ospedaliero‐Universitaria di Bologna Bologna Italy; ^9^ Division of Pathology IRCCS Azienda Ospedaliero‐Universitaria di Bologna Bologna Italy; ^10^ Cardiologic Center University of Ferrara Ferrara Italy; ^11^ Maria Cecilia Hospital, GVM Care & Research Cotignola Italy

**Keywords:** Cardiomyopathy, Echocardiography, Prognosis, Progression, Restrictive

## Abstract

**Aims:**

Transthyretin amyloid cardiomyopathy (ATTR‐CM) is an increasingly diagnosed disease. Echocardiography is widely utilized, but studies to confirm the value of echocardiography for tracking changes over time are not available. We sought to describe (i) changes in multiple echocardiographic parameters; (ii) differences in rate of progression of three predominant genotypes; and (iii) the ability of changes in echocardiographic parameters to predict prognosis.

**Methods and results:**

We prospectively studied 877 ATTR‐CM patients attending our centre between 2000 and 2020. Serial echocardiography findings at baseline, 12 months and 24 months were compared with survival. Overall, 565 patients had wild‐type ATTR‐CM and 312 hereditary ATTR‐CM (201 with V122I; 90 with T60A). There was progressive worsening of structural and functional parameters over time, patients with V122I ATTR‐CM showing more rapid worsening of left and right ventricular structural and functional parameters compared to both wild‐type and T60A ATTR‐CM. Among a wide range of echocardiographic analyses, including deformation‐based parameters, only worsening in the degree of mitral (MR) and tricuspid regurgitation (TR) at 12‐ and 24‐month assessments was associated with worse prognosis (change at 12 months: MR, hazard ratio 1.43 [95% confidence interval 1.14–1.80], *p* = 0.002; TR, hazard ratio 1.38 [95% confidence interval 1.10–1.75], *p* = 0.006). Worsening in MR remained independently associated with poor prognosis after adjusting for known predictors.

**Conclusion:**

In ATTR‐CM, echocardiographic parameters progressively worsen over time. Patients with V122I ATTR‐CM demonstrate the most rapid deterioration. Worsening of MR and TR were the only parameters associated with mortality, MR remaining independent after adjusting for known predictors.

## Graphical abstract

Central role of mitral and tricuspid regurgitation in transthyretin amyloid cardiomyopathy (ATTR‐CM) disease progression. Amyloid valve infiltration leads to worsening mitral and tricuspid valve insufficiency identified on serial echocardiography with associated evidence of valvular dysfunction and remodelling on histology. Worsening in mitral (MR) and tricuspid regurgitation (TR) is independently associated with mortality at 12 and 24 months.

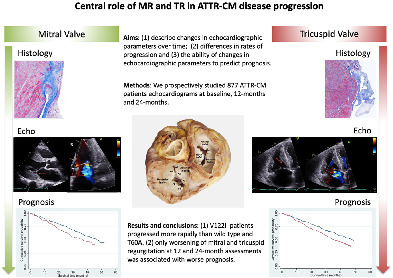

## Introduction

Transthyretin amyloid cardiomyopathy (ATTR‐CM) is a progressive and usually fatal disease caused by accumulation of ATTR amyloid deposits between cells in the myocardium. ATTR‐CM is classified as hereditary (hATTR‐CM) when associated with mutations in the transthyretin (*TTR*) gene, or more frequently as non‐hereditary (wtATTR‐CM) when the gene is wild‐type. Diagnosis of ATTR‐CM has lately increased dramatically reflecting use of non‐biopsy imaging techologies coupled with improved awareness and development of specific therapies.^1^ However, the natural history remains poorly understood as are the most clinically meaningful parameters acquired by serial echocardiography, which is nevertheless performed routinely to monitor disease progression. Blood biomarker studies to date have mainly focused on wtATTR‐CM.^2^

Echocardiography typically offers the first clues to diagnosis of ATTR‐CM and its prognosis.^3^, ^4^, ^5^, ^6^, ^7^ However, whilst its value for diagnosis and prognosis at patients' initial presentation has been established,^8^ the role of serial echocardiography to ascertain and elucidate the basis for disease progression within individuals has been little studied. To date, most such data have been generated from retrospective analyses of small cohorts or sub‐analyses of large multicentre clinical trials focused on identifying differences between study arms rather than evaluating changes within individual patients.^9^, ^10^, ^11^, ^12^, ^13^, ^14^ The need to identify and validate imaging parameters that inform the latter was recently highlighted in expert consensus guidance, which acknowledged the current lack of hard data.^15^

The aims of this study were (i) to describe serial changes in structural and functional echocardiographic parameters across large populations with wtATTR and hATTR‐CM; (ii) to compare rates of disease progression in wtATTR‐CM and two leading forms of hATTR‐CM; and (iii) to assess the ability of changes in echocardiographic parameters to predict prognosis.

## Methods

The National Amyloidosis Centre (NAC) is the centrally commissioned single centre in the United Kingdom for the diagnosis and monitoring of amyloidosis. Therefore patients seen at the NAC are likely to represent the national caseload in the UK. Patients referred to the NAC, Royal Free Hospital, London, UK between 2000 and 2020 in whom ATTR‐CM was confirmed using validated diagnostic criteria were invited to participate in a prospective protocolized clinical follow‐up programme comprising serial clinical assessments and systematic evaluation of cardiac parameters. This was a study of consecutive patients diagnosed with ATTR‐CM. Patients who did not attend at 12‐month follow‐up, but did attend at 24‐month follow‐up were included in the study. Briefly, the diagnosis of ATTR‐CM was established on the basis of presence of symptoms of heart failure (HF) together with a characteristic echocardiogram and either: direct endomyocardial biopsy (EMB) proof of ATTR amyloid or presence of ATTR amyloid in an extra‐cardiac biopsy along with cardiac uptake on ^99m^Technetium labelled 3,3‐diphosphono‐1,2‐propanodicarboxylic acid (^99m^Tc‐DPD) scintigraphy; or Perugini grade 2 or 3 cardiac uptake on ^99m^Tc‐DPD scintigraphy in the absence of an abnormal serum free light chain ratio and monoclonal immunoglobulin in the serum and urine by immunofixation.^16^ All patients underwent sequencing of the *TTR* gene. Patients were managed in accordance with the Declaration of Helsinki and provided written informed consent for retrospective analysis and publication of their data ( Royal Free Ethics Committee, UK: ref: 06/Q0501/42). As part of the study, histological analysis of explanted whole hearts was performed at the University of Bologna (AOU of IRCCS Azienda Ospedaliero‐Universitaria di Bologna, Italy:79/2014/U/Sper).

### Echocardiography

All two‐dimensional (2D) transthoracic echocardiograms were performed by experienced operators blinded to the final outcome using GE Vivid E9 ultrasound machine equipped with a 5S probe and measurements were analysed according to current recommendations of non‐invasive assessment of native valvular regurgitation^17^ (online supplementary *Appendix* *S1* for echocardiographic methods). The echocardiograms were not re‐analysed for this study, but the values simply entered from the clinical database.

Mitral regurgitation (MR) severity was graded, using a multiparametric approach as none (grade 0) in absence of any detectable regurgitant jet, and definitely mild (grade 1) and definitely severe (grade 5). The current recommendations^17^ were adapted to our population and in case of intermediate values in keeping with probable moderate regurgitation, semiquantitative and quantitative parameters and supporting signs were used, resulting in a further subclassification into more nuanced grading of regurgitation defined as mild‐moderate (grade 2), moderate (grade 3) and moderate‐severe (grade 4).^17^ The mechanism of MR was described and classified as primary (organic), secondary (functional) and mixed when both components are present. Anteroposterior mitral annulus (AP‐MA) dimensions in end‐diastole were evaluated in the conventional parasternal long‐axis view and dilatation was defined by an end‐diastolic diameter ≥35 mm.^17^

Tricuspid regurgitation (TR) severity was graded, using a multiparametric approach, as none (grade 0) in absence of any detectable regurgitant jet, and definitely mild (grade 1) and severe (grade 5) when the standard criteria are met. When in the range of probable moderate TR, a further subclassification into mild‐moderate (grade 2), moderate (grade 3) and moderate‐severe (grade 4) was adopted. The mechanism of TR was described and classified as primary (organic), secondary (functional) and mixed when both components are present. With 2D echocardiography the three leaflets of tricuspid valve (TV) cannot always be visualized simultaneously, therefore we focused our analysis on the conventional apical four‐chamber (A4Ch) view where the septal leaflet is adjacent to the septum and the leaflet adjacent to the right ventricular (RV) free wall could be anterior or posterior.^17^ Tricuspid annulus dimensions were measured in end‐diastole in the conventional A4Ch view and dilatation was defined by an end‐diastolic diameter ≥40 mm.^17^

Two separate analyses were performed using progression of ‘at least’ one grade of valvular regurgitation and using progression of ‘at least’ two grades of valvular regurgitation. For example, a patient who progresses from mild (grade 1) to mild‐to‐moderate MR/TR and above (grade 2 and above) will be classified into progression of ‘at least’ one grade of valvular regurgitation and a patient who progresses from mild (grade 1) to moderate and above (grade 3 and above) will be classified into progression of ‘at least’ two grades of valvular regurgitation.

### Histology

Two explanted hearts were evaluated using standards and definitions proposed by the Committee of the Society for Cardiovascular Pathology and the Association for European Cardiovascular Pathology.^18^ After adequate fixation in 10% formaldehyde, the hearts were cut in parallel transverse sections, 1.0 to 1.5 cm thick, from the apex to approximately 4 cm below the atrio‐ventricular (AV) groove. The base of the heart was then cut along the longitudinal axis through the AV valves. Wide sampling for histology included specimens from both atria; right ventricle, left ventricle and septum at basal, medium (an entire midventricular section) and apical levels; major subepicardial coronary arteries; AV valves.

Specifically, the anterior and posterior leaflets of AV valves were obtained using a longitudinal section including the annulus, the atrial and ventricular myocardium, the leaflets with the chordae tendinae and the papillary muscles. From the paraffin blocks 2 μm thick sections were cut for histological analysis and stained with haematoxylin–eosin, which characterizes amyloid as a homogeneous, slightly eosinophilic, amorphous and unstructured substance, with Mallory trichrome, which stains amyloid pinkish grey, collagen dark/bright blue and myocytes red, and Congo red which highlights amyloid with the typical apple green birefringence when observed under polarized light.

### Statistical analysis

All mortality data were obtained via the UK Office of National Statistics. The mortality endpoint was defined as time to death from baseline for all deceased patients and time to censor date, 13 March 2020, from baseline among the remainder. Follow‐up was restricted to ≤60 months from baseline, after which patients were censored due to the low number of patients at risk after 60 months. Patients who were enrolled in clinical trials or who initiated disease‐modifying therapy were censored on the date that they were enrolled or started treatment. The three genotypic subgroups of interest were wild‐ wtATTR‐CM, V122I‐associated hATTR‐CM (V122I‐hATTR‐CM), and T60A‐associated hATTR‐CM (T60A‐hATTR‐CM). Summary data are presented as mean and standard deviation (SD) for numerical variables, all of which were approximately normally distributed. Paired *t*‐tests were used for numerical variables to compare changes in variables between different time points; these changes were approximately normally distributed. Linear regression analysis was used for all the numerical outcome variables at 12 and 24 months to allow a comparison of the genotypes after adjusting for age at baseline and baseline value of the variable. The assumptions underlying the regression analyses were verified by a study of the residuals. Survival was evaluated with Cox proportional hazards regression analysis, providing estimated hazard ratios (HRs) with 95% confidence intervals (CIs) and Kaplan–Meier curves. The proportional hazards assumption was verified for all survival analyses. Landmark analyses were performed for the change in echocardiographic variables at 12 months and 24 months, using initially 12 and then 24 months as the starting point. Furthermore, 14 echocardiographic parameters and a validated staging system that uses estimated glomerular filtration rate (eGFR) and N‐terminal pro‐hormone B‐type natriuretic peptide (NT‐proBNP)^19^ were entered into a multivariable Cox proportional hazards regression. The regression analysis was carried out as a non‐stepwise analysis and the parameters were selected a priori based upon clinical relevance and statistical significance in univariable analyses. The model incorporated echocardiographic variables including severe aortic stenosis, degree of MR and TR, stroke volume (SV) index, E/e′ average, right atrial area (RAA) index, left atrial area (LAA) index, left ventricular longitudinal strain (LV LS), ejection fraction (EF) and tricuspid annular plane systolic excursion (TAPSE) at baseline and MR and TR progression of ‘at least’ one grade, change in SV index and change in RAA index from baseline to 12 months. To assess the reproducibility of the degree of MR and TR and the reproducibility of the visual assessment of the mitral and tricuspid valves, two observers measured each of the variables on 100 patients blinded. The weighted kappa was calculated and assessed according to the Landis and Koch classification. A significance level of 0.05 was adopted for all hypothesis tests and the data were analysed using SPSS (IBM Corp., released 2020; IBM SPSS Statistics for Windows, Version 27.0; IBM Corp, Armonk, NY, USA) for every analysis apart from the survival analyses when Stata (StataCorp. 2019. Stata Statistical Software: release 16; StataCorp LLC, College Station, TX, USA) was used.

## Results

### Characteristics of the cohort at baseline

Of 1240 patients studied at baseline, 877 underwent echocardiography at 12‐ and 24‐month timepoints (843 and 612, respectively) and were included in this study. Thirty‐four patients had only baseline and 24‐month echocardiograms (online supplementary *Figure* *S1*). Mean follow‐up times were 12.50 months (SD 2.04) and 24.50 months (SD 2.73). Of the 877 patients, 565 (64.4%) had wtATTR‐CM (mean age 76.71, SD 6.67 years; 95.2% male); 201 (22.9%) had V122I‐hATTR‐CM (mean age 75.28, SD 7.17 years; 71.1% male); 90 (10.3%) had T60A‐hATTR‐CM (mean age 66.13, SD 6.49; 72.2% male); and 21 (2.4%) had hATTR‐CM associated with other mutations (mean age 61.71, SD 10.64 years; 81% male). Of the 877 patients, 195 (22%) were Afro‐caribbean.

### Disease progression in ATTR amyloidosis

At 12 and 24 months in the overall population, there was evidence of progressive LV concentric remodelling with a significant increase in wall thickness and reduction in LV cavity size, accompanied by atrial enlargement (Table 1). There was an observed mean reduction in parameters of systolic function including SV and mitral annular plane systolic excursion (MAPSE), as well as worsening of parameters of diastolic function including septal and lateral e′ velocities. Additionally, there was a significant progressive deterioration in deformation‐based parameters including LV LS and in volumetric measures of function such as myocardial contraction fraction (MCF). There was also significant worsening in right heart structure and function characterized by deterioration in TAPSE and tissue Doppler RV S' velocity, RV LS, increase in pulmonary artery systolic pressures and RV to pulmonary circulation uncoupling (*Table* *1*).

**Table 1:** Echocardiographic findings in patients with transthyretin amyloid cardiomyopathy at baseline, 12 and 24 months

Echocardiographic variables	Baseline (*n* = 877)	12 months (*n* = 843)	24 months (*n* = 612)
IVSd (mm)	16.87 (2.37)	17.22 (2.35)*	17.55 (2.33)**
PWTd (mm)	16.30 (2.47)	16.80 (2.35)*	17.19 (2.39)**
LVM (g)	313.90 (82.07)	319.30 (83.88)*	327.96 (87.29)**
LVEDD (mm)	43.74 (5.60)	43.02 (5.79)*	42.78 (5.82)**
LVEDDi (mm/m^2^)	23.11 (2.94)	22.72 (3.02)*	22.53 (3.03)**
MWT (mm)	16.58 (2.29)	17.01 (2.24)*	17.37 (2.24)**
LVEDV (ml)	76.53 (24.59)	77.31 (27.10)	75.78 (25.41)
LVEDVi (ml/m^2^)	40.15 (11.96)	40.48 (13.24)	39.61 (12.32)
LVESV (ml)	39.94 (17.26)	40.59 (18.43)	41.82 (18.08)**
LVESVi (ml/m^2^)	20.94 (8.67)	21.22 (9.24)	21.86 (9.05)**
SV (ml)	36.59 (12.55)	35.82 (14.52)	33.95 (12.94)**
SV index (ml/m^2^)	19.22 (6.16)	18.78 (7.13)	17.75 (6.38)**
EF (%)	48.66 (10.52)	47.74 (11.94)*	45.65 (11.15)**
LAA (cm^2^)	26.22 (5.42)	26.47 (5.51)	26.64 (5.31)**
LAA index (cm^2^/m^2^)	13.87 (2.93)	13.98 (2.89)	14.02 (2.73)**
RAA (cm^2^)	24.05 (6.14)	24.56 (6.44)*	25.16 (6.80)**
RAA index (cm^2^/m^2^)	12.67 (3.09)	12.94 (3.29)*	13.21 (3.45)**
E/A	2.13 (1.09)	2.13 (1.01)*	2.12 (1.02)**
DT (ms)	182.04 (56.16)	177.58 (55.29)*	174.25 (50.71)**
e′ lateral (cm/s)	6.34 (2.11)	6.15 (2.13)*	5.80 (2.25)**
e′ septal (cm/s)	4.53 (1.52)	4.38 (1.52)*	4.17 (1.58)**
E/e′ lateral	14.78 (5.97)	15.52 (6.63)*	16.78 (7.56)**
E/e′ average	16.78 (6.04)	17.56 (6.54)*	18.72 (7.31)**
MAPSE (mm)	8.18 (2.58)	7.81 (2.56)*	7.42 (2.49)**
TAPSE (mm)	15.34 (4.61)	14.35 (4.69)*	13.53 (4.57)**
TAPSE/PASP	0.40 (0.18)	0.37 (0.20)	0.34 (0.17)**
S′ tricuspid (cm/s)	10.45 (3.08)	9.89 (3.18)*	9.27 (3.02)**
RWT	0.76 (0.16)	0.80 (0.17)*	0.82 (0.17)**
MCF (%)	0.16 (0.06)	0.15 (0.05)*	0.14 (0.05)**
LV LS (%)	–11.17 (3.71)	–10.15 (3.84)*	–9.45 (3.73)**
RV LS (%)	–12.71 (3.99)	–11.74 (3.81)*	–11.11 (3.82)**
SABr	5.39 (7.01)	5.93 (6.54)*	5.49 (7.38)
RALS	1.77 (1.27)	1.74 (2.41)	2.02 (2.33)**
PASP (mmHg)	40.51 (10.21)	40.96 (10.54)	41.89 (10.74)**
LA strain reservoir	12.17 (8.67)	10.93 (8.97)*	9.68 (7.93)**
LA strain contraction	5.51 (4.98)	5.57 (5.86)	4.75 (5.02)**

Data are presented as means (standard deviation).

DT, deceleration time; EF, ejection fraction; IVSd, interventricular septum in diastole; LA, left atrial; LAA, left atrial area; LS, longitudinal strain; LVEDD, left ventricular end‐diastolic diameter; LVEDDi, left ventricular end‐diastolic diameter index; LVEDV, left ventricular end‐diastolic volume; LVEDVi, left ventricular end‐diastolic volume index; LVESV, left ventricular end‐systolic volume; LVESVi, left ventricular end‐systolic volume index; LVM, left ventricular mass; MAPSE, mitral annular plane systolic excursion; MCF, myocardial contraction fraction; MWT, mean wall thickness; PASP, pulmonary artery systolic pressure; PWTd, posterior wall thickness in diastole; RAA, right atrial area; RALS, relative apical longitudinal strain; RV, right ventricular; RWT, relative wall thickness; SABr, systolic apex to base ratio; SV, stroke volume; TAPSE, tricuspid annular plane systolic excursion.

*P*‐values for paired *t*‐tests: *p < 0.05 for baseline vs. 12 months (*n* = 843); **for baseline vs. 24 months (*n* = 612). Statistical significance is represented by *p*‐values < 0.05.

### Differential disease progression in wtATTR‐CM, V122I‐hATTR‐CM and T60A‐hATTR‐CM

When compared to wtATTR‐CM, after adjusting for differences in baseline parameters and age, patients with V122I‐hATTR‐CM showed significantly more rapid disease progression at 12 and 24 months, with more rapid deterioration in LV systolic function parameters including SV, EF, MCF, LV LS and MAPSE. There was also significantly more rapid deterioration in tissue Doppler e′ lateral and septal annular velocities and worsening in diastolic parameters such as deceleration time (DT) and E/A ratio. There was more rapid and marked concentric remodelling in V122I‐hATTR‐CM compared to wtATTR‐CM, with smaller LV cavity sizes and greater relative wall thickness. There was also more rapid deterioration in right heart structure and function in patients with V122I‐hATTR‐CM compared to wtATTR‐CM, characterized by deterioration in tissue Doppler RV S' velocity. In contrast, after adjusting for baseline parameters and age at baseline, at 12 and 24 months, patients with T60A‐hATTR‐CM had significantly less rapid progression in parameters of LV systolic dysfunction such as EF, and in diastolic parameters such as DT and E/A ratio than those with wtATTR‐CM. There was also comparatively less rapid deterioration in parameters of right heart structure and function including TAPSE and RV LS in patients with T60A‐hATTR‐CM compared to wtATTR‐CM (*p*‐values for all comparisons < 0.05) (online supplementary *Tables* *S1* and *S2*).

### Change in echocardiographic parameters and prognosis

Of the 877 patients studied at baseline, at mean follow‐up of 40.4 months (SD 14.8), 349 (39.8%) of 877 patients had died, including 209 (37%) in the wtATTR‐CM group, 104 (51.7%) in the V122I‐hATTR‐CM group, 29 (32.2%) in the T60A‐hATTR‐CM, and 7 (33.3%) in the non‐T60A non‐V122I group.

The association between the change at 12 and 24 months in 27 echocardiographic variables and prognosis was explored by univariable Cox regression analysis (*Table* *2*). For these landmark analyses, the mean follow‐up was 27.8 months (SD 14.9) and 20.6 months (SD 11.7) from 12 and 24‐month echocardiographic follow‐up, respectively. At 12 months, a decrease in SV index was associated with mortality (HR 0.98, 95% CI 0.97–1.00, *p* = 0.019). At both timepoints of 12 and 24 months, the increase in RAA index was independently associated with mortality (HR 1.04, 95% CI 1.00–1.09, *p* = 0.050 and HR 1.07, 95% CI 1.01–1.12, *p* = 0.013, respectively).

**Table 2:** Univariable Cox regression analysis of risk of mortality using change in echocardiographic parameters

Echocardiographic variable	Baseline to 12 months		Baseline to 24 months	
	HR (95% CI)	*p*‐value	HR (95% CI)	*p*‐value
SV index (ml/m^2^)	0.98 (0.97–1.00)	**0.019**	0.98 (0.96–1.00)	0.088
EF (%)	0.99 (0.98–1.00)	0.201	0.99 (0.97–1.00)	0.133
MCF (%)	0.57 (0.06–5.47)	0.624	0.29 (0.02–4.49)	0.374
LAA index (cm^2^/m^2^)	0.97 (0.93–1.01)	0.187	0.98 (0.93–1.03)	0.411
RAA index (cm^2^/m^2^)	1.04 (1.00–1.09)	**0.050**	1.07 (1.01–1.12)	**0.013**
IVSd index (mm/m^2^)	1.15 (0.96–1.39)	0.126	1.13 (0.94–1.37)	0.188
MR progression ‘at least’ 1 grade	1.43 (1.14–1.80)	**0.002**	1.34 (1.01–1.77)	**0.043**
MR progression ‘at least’ 2 grades	1.83 (1.31–2.55)	**0.000**	1.49 (1.01–2.19)	**0.043**
TR progression ‘at least’ 1 grade	1.38 (1.10–1.75)	**0.006**	1.42 (1.08–1.86)	**0.013**
TR progression ‘at least’ 2 grades	1.50 (1.10–2.05)	**0.011**	1.55 (1.08–2.22)	**0.019**
LV LS (%)	0.99 (0.95–1.02)	0.409	1.00 (0.96–1.04)	0.977
RV LS (%)	0.99 (0.96–1.03)	0.653	1.02 (0.98–1.06)	0.330
SABr	1.00 (0.99–1.01)	0.743	0.99 (0.98–1.01)	0.423
RALS	0.98 (0.93–1.03)	0.378	1.00 (0.94–1.06)	0.959
E/A	1.00 (0.85–1.19)	0.964	0.98 (0.78–1.22)	0.838
DT (ms)	1.00 (0.99–1.00)	0.728	1.00 (0.99–1.00)	0.889
e′ lateral (cm/s)	0.98 (0.92–1.04)	0.510	1.01 (0.95–1.08)	0.662
e′ septal (cm/s)	0.99 (0.92–1.07)	0.799	0.97 (0.89–1.06)	0.492
E/e′ lateral	1.00 (0.98–1.02)	0.891	0.98 (0.96–1.01)	0.159
E/e′ average	1.00 (0.98–1.02)	0.992	0.98 (0.95–1.00)	0.105
MAPSE (mm)	1.01 (0.97–1.05)	0.667	0.98 (0.93–1.03)	0.348
TAPSE (mm)	1.00 (0.97–1.03)	0.972	0.98 (0.95–1.01)	0.143
PASP (mmHg)	1.00 (0.99–1.01)	0.586	1.00 (0.99–1.02)	0.563
TAPSE/PASP	1.10 (0.59–2.04)	0.773	0.81 (0.37–1.78)	0.595
RWT	1.17 (0.45–3.06)	0.744	1.22 (0.40–3.76)	0.725
LA strain reservoir	1.00 (1.00–1.02)	0.622	1.01 (0.99–1.03)	0.561
LA strain contraction	1.02 (0.98–1.05)	0.449	0.99 (0.93–1.05)	0.647

CI, confidence interval; DT, deceleration time; EF, ejection fraction; HR, hazard ratio; IVSd, interventricular septum in diastole; LA, left atrial; LAA, left atrial area; LS, longitudinal strain; LV, left ventricular; LVEDD, left ventricular end diastolic diameter; LVEDV, left ventricular end diastolic volume; LVESV, left ventricular end systolic volume; LVM, left ventricular mass; MAPSE, mitral annular plane systolic excursion; MCF, myocardial contraction fraction; MR, mitral regurgitation; MWT, mean wall thickness; PASP, pulmonary artery systolic pressure; PWTd, posterior wall thickness in diastole; RAA, right atrial area; RALS, relative apical longitudinal strain; RV, right ventricular; RWT, relative wall thickness; SABr, systolic apex to base ratio; SV, stroke volume; TAPSE, tricuspid annular plane systolic excursion; TR, tricuspid regurgitation.

*Note:* Univariable Cox regression analysis of risk of mortality using change in echocardiographic parameters from baseline to 12 months, and baseline to 24 months. Statistical significance, shown in bold, is represented by *p*‐values < 0.05.

**Figure 1:**
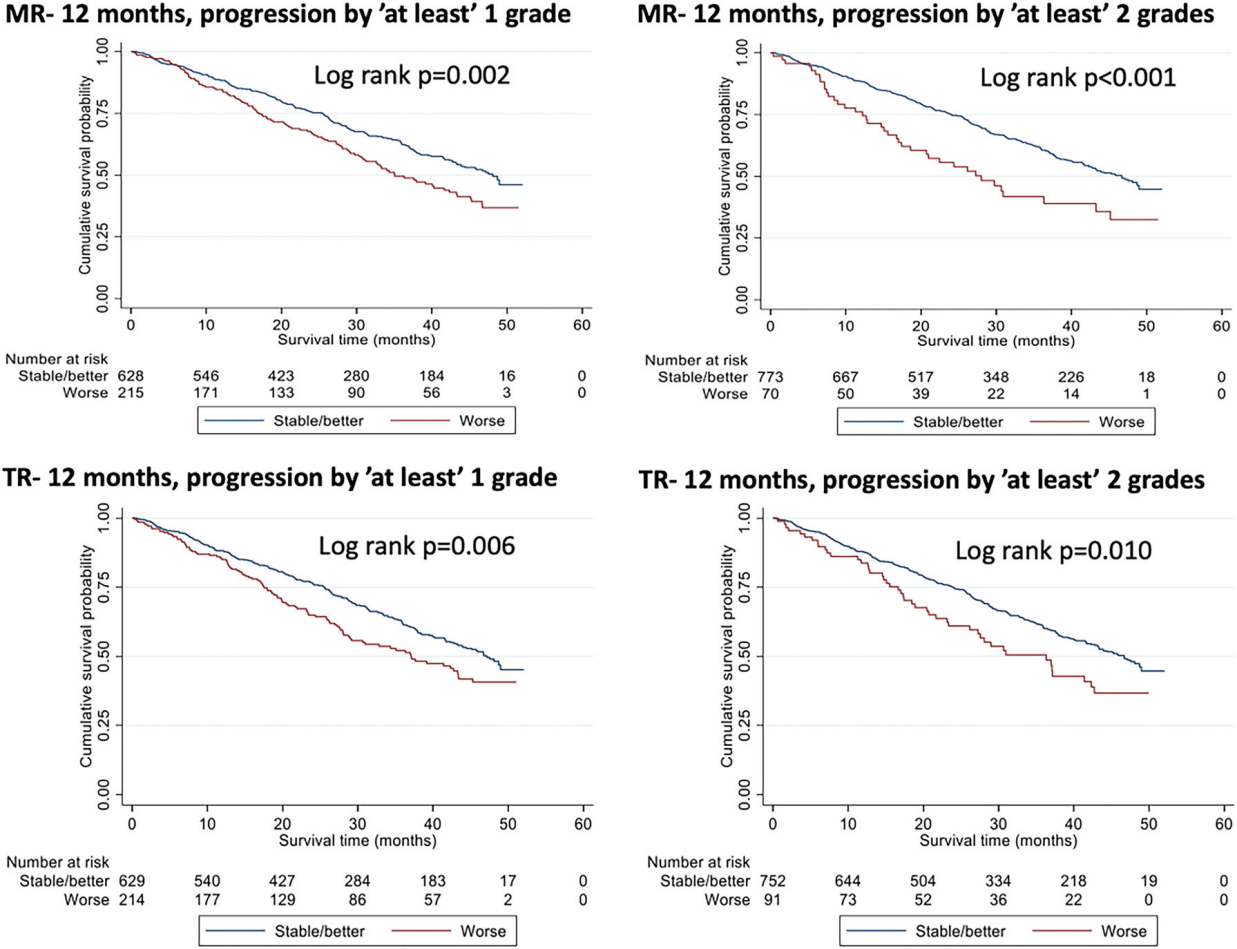
Kaplan–Meier curves of mitral (MR) and tricuspid regurgitation (TR) progression from baseline to 12 months showing progression of ‘at least’ one or two grades.

**Figure 2:**
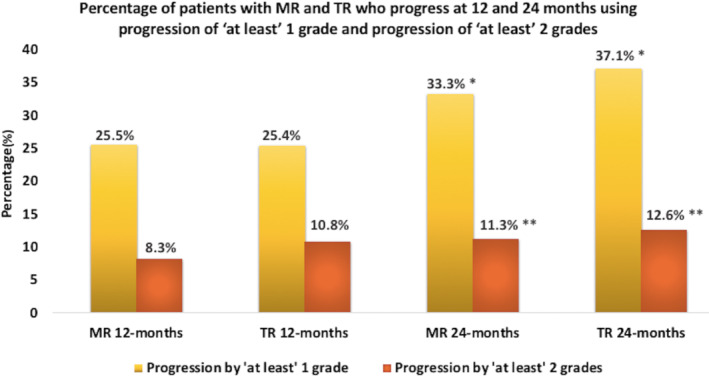
Patients with mitral (MR) and tricuspid regurgitation (TR) with progression. Percentage of patients with MR and TR who progress at 12 and 24 months using progression of ‘at least’ one grade and progression of ‘at least’ two grades. *p = 0.001 for MR progression of ‘at least’ one grade from 12 to 24 months; *p* < 0.001 value for TR progression of ‘at least’ one grade from 12 to 24 months. **p = 0.058 for MR progression of ‘at least’ two grades from 12 to 24 months; *p* = 0.319 for TR progression of ‘at least’ two grades from 12 to 24 months.

At both time points, using progression of ‘at least’ one grade and progression of ‘at least’ two grades, a worsening in the degree of MR and TR was predictive of mortality (all *p*‐values < 0.05; *Table* *2*). Kaplan–Meier curves displaying the prognostic impact of worsening in the degree of MR and TR at 12 months for progression of ‘at least’ one grade and progression by ‘at least’ two grades are represented in *Figure* *1* (see *Figure* *2*, *Table* *3* and online supplementary *Appendix* *S1* for valvular severity and progression at all timepoints).

**Table 3:** Patients with mitral and tricuspid regurgitation at baseline, 12 and 24 months

	Baseline (*n* = 877)	12 months (*n* = 843)	24 months (*n* = 612)
MR grade			
None	293 (33.4%)	280 (33.2%)	172 (28.1%)
Mild	310 (35.3%)	282 (33.5%)	251 (41%)
Mild/moderate	161 (18.4%)	152 (18%)	105 (17.2%)
Moderate	104 (11.9%)	111 (13.2%)	74 (12.1%)
Moderate/severe	8 (0.9%)	13 (1.5%)	8 (1.3%)
Severe	1 (0.1%)	5 (0.6%)	2 (0.3%)
TR grade			
None	312 (35.6%)	290 (34.4%)	184 (30.1%)
Mild	289 (33)	248 (29.4%)	212 (34.6%)
Mild/moderate	124 (14.1)	129 (15.3%)	95 (15.5%)
Moderate	110 (12.5)	121 (14.4%)	85 (13.9%)
Moderate/severe	22 (2.5)	24 (2.8%)	17 (2.8%)
Severe	20 (2.3)	31 (3.7%)	19 (3.1%)

MR, mitral regurgitation; TR, tricuspid regurgitation.

Fourteen echocardiographic parameters and a validated staging system that uses eGFR and NT‐proBNP^19^ was entered into a multivariable Cox proportional hazards regression. The model incorporated echocardiographic variables including severe aortic stenosis, degree of MR and TR, SV index, E/e′ average, RAA index, LAA index, LV LS, EF and TAPSE at baseline and MR and TR progression of ‘at least’ one grade, change in SV index and change in RAA index from baseline to 12 months. Fifty‐seven patients at baseline had aortic stenosis, of which nine patients had severe aortic stenosis. The results demonstrated that worsening in MR retained independent prognostic significance together with degree of MR at baseline, presence of severe aortic stenosis at baseline, SV index at baseline but also SV index change over time (*p* < 0.05 for all) (see *Table* *4* for multivariable model).

**Table 4:** Multivariable model

Variables	HR (95% CI)	*p*‐value
NAC stage baseline^a^		
Stage 2	1.31 (0.99–1.73)	0.057
Stage 3	2.31 (1.64–3.25)	**<0.001**
MR baseline^b^		
Grade 1	1.42 (1.01–2.01)	**0.045**
Grade 2	1.69 (1.12–2.54)	**0.012**
Grade 3	2.10 (1.32–3.35)	**0.002**
Grade 4	2.50 (1.01–6.20)	**0.047**
Grade 5	8.15 (1.01–65.66)	**0.049**
TR baseline^c^		
Grade 1	1.07 (0.76–1.51)	0.682
Grade 2	1.02 (0.66–1.58)	0.932
Grade 3	1.11 (0.70–1.76)	0.646
Grade 4	1.00 (0.48–2.09)	0.997
Grade 5	2.26 (1.13–4.52)	**0.021**
Severe AS baseline^d^	5.11 (1.81–14.45)	**0.002**
SV index baseline (ml/m^2^)	0.96 (0.93–0.99)	**0.006**
RAA index baseline (cm^2^/m^2^)	1.02 (0.96–1.08)	0.481
LAA index baseline (cm^2^/m^2^)	1.02 (0.97–1.07)	0.454
E/e′ average baseline	1.00 (0.98–1.03)	0.662
LS (%) baseline	1.03 (0.98–1.08)	0.218
EF (%) at baseline	0.99 (0.97–1.00)	0.061
TAPSE (mm) at baseline	1.00 (0.97–1.04)	0.863
MR ‘progression of at least one grade’ at 12 months^e^	1.51 (1.14–2.01)	**0.004**
TR ‘progression of at least one grade’ at 12 months^e^	1.04 (0.77–1.40)	0.785
SV index change (ml/m^2^) at 12 months	0.97 (0.95–0.99)	**0.002**
RAA index change (cm^2^/m^2^) at 12 months	1.03 (0.98–1.09)	0.184

AS, aortic stenosis; CI, confidence interval; EF, ejection fraction; HR, hazard ratio; LAA, left atrial area; LS, longitudinal strain; MR, mitral regurgitation; NAC, National Amyloidosis Centre; RAA, right atrial area; SV, stroke volume; TAPSE, tricuspid annular plane systolic excursion; TR, tricuspid regurgitation.

^a^ NAC stage 1 is the reference category.

^b^ MR grade 0 is the reference category.

^**c**^ TR grade 0 is the reference category.

^d^ Absence of severe AS is the reference category.

^e^ Absence of ‘progression of at least one grade’ is the reference category.

Multivariable Cox proportional hazards regression analysis. Statistical significance, shown in bold, is represented by *p* < 0.05.

### Structural abnormalities in mitral and tricuspid valves

**Figure 3:**
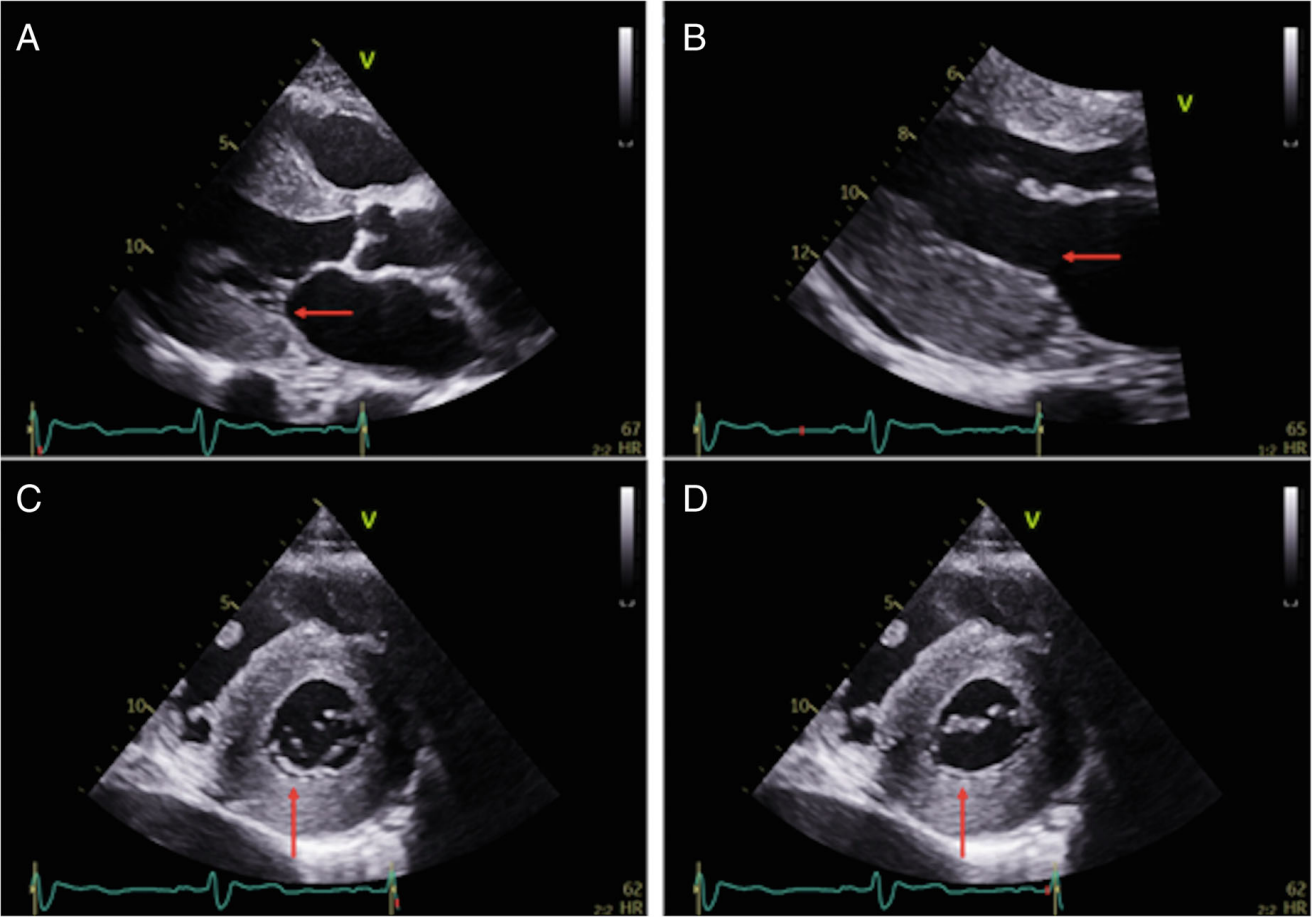
Transthoracic echocardiogram in a patient with transthyretin amyloid cardiomyopathy: mitral valve. A 78‐year‐old lady with V122I‐associated hereditary transthyretin amyloidosis. (*A*) Two‐dimensional transthoracic echocardiographic parasternal long‐axis view of the mitral valve with both thickened and markedly shortened posterior mitral leaflet (red arrow). (B) Zoomed parasternal long‐axis view of the mitral valve in diastole where the posterior mitral leaflet appears to be completely ‘disappeared’ (red arrow). (*C*) Short‐axis view of the mitral valve in systole, confirming a short posterior mitral leaflet (red arrow) in early systole with eccentric posterior coaptation line. (*D*) Short‐axis view of the mitral valve in diastole, demonstrating the ‘disappeared’ posterior mitral leaflet as fixed and englobed into the posterior wall (red arrow).

**Figure 4:**
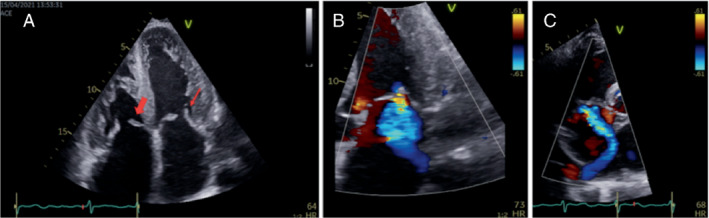
Transthoracic echocardiogram in a patient with transthyretin amyloid cardiomyopathy: mitral and tricuspid valves. A 69‐year‐old lady with hereditary transthyretin amyloidosis associated with V122I TTR variant. (*A*) Two‐dimensional transthoracic echocardiographic four‐chamber view of both atrio‐ventricular valves, markedly thickened. Septal tricuspid valve leaflet (thick red arrow) and posterior mitral valve leaflet (thin red arrow) also appear restricted (reduced mobility). (*B*) Colour Doppler of moderate mitral regurgitation, posteriorly directed due to thickened and restricted posterior mitral valve leaflet. (*C*) Colour Doppler of extremely eccentric tricuspid regurgitation due to thickened and restricted septal tricuspid valve leaflet.

Baseline visual assessment of the mitral and tricuspid valves by echocardiography is described in *Table* *5* and valvular abnormalities by echocardiography are illustrated in *Figures* *3* and *4*. To assess the reproducibility of the degree of MR and TR, two observers measured each of the variables on 100 patients blinded. The weighted kappa was 0.824 (95% CI 0.784–0.887) for MR and 0.824 (0.784–0.887) for TR, in each case indicating almost perfect agreement according to the Landis and Koch classification. Similarly, to assess the reproducibility of visual assessment of the mitral and tricuspid valves, two observers assessed 100 patients blinded. The weighted kappa was 0.520 (95% CI 0.324–0.717) for posterior mitral valve leaflet (PMVL) thickness, 0.943 (95% CI 0.877–1.009) for PMVL length, 0.976 (95% CI 0.930–1.023) for PMVL restriction, 0.818 (95% CI 0.732–0.905) for anterior mitral valve leaflet (AMVL) restriction, 0.854 (95% CI 0.812–0.896) for mitral annulus dimensions, 0.740 (95% CI 0.574–0.907) for mitral valve papillary muscle thickness, 0.868 (95% CI 0.767–0.970) for septal tricuspid leaflet thickness, 0.834 (95% CI 0.740–0.928) for septal tricuspid leaflet restriction, 0.811 (95% CI 0.703–0.919) for non‐septal tricuspid leaflet restriction and 0.854 (95% CI 0.813–0.894) for tricuspid annulus dimensions, indicating overall good agreement according to the Landis and Koch classification.

**Table 5:** Structural abnormalities in mitral and triscuspid valves by echocardiography

**Mitral valve, *n* = 877**	
Leaflets	
Diffuse thickening of PMVL	818 (93.3%)
Restriction of PMVL	717 (81.8%)
Shortened PMVL with a restricted base and ‘englobed’ appearance into adjacent infiltrated basal lateral wall	595 (67.8%)
PMVL hidden, appearing completely ‘englobed’ into the myocardium	96 (10.9%)
Restriction of AMVL	458 (52.2%)
Sub‐valvular apparatus	
Prominent or markedly thickened papillary muscles and chordae tendinae	724 (82.6%)
MV annulus	
MV annulus dilatation	27 (3.1%)
Mean mitral annulus diameter (mm)	27.07 (3.98)
Regurgitant jet	
Eccentric	206 (23.5%) (21.4% towards the lateral atrial wall, 2.1% anteriorly directed)
Central	346 (39.5%)
**Tricuspid valve, *n* = 877**	
Leaflets	
Diffuse thickening of the TV leaflets	716 (81.6% )
Restriction of TVSL	413 (47.1%)
Thickening and restriction of non‐septal leaflet	252 (28.7%)
TV annulus	
TV annulus dilatation	144 (16.4%)
Mean tricuspid annulus diameter (mm)	33.8 (6.2)
Regurgitant jet	
Eccentric	229 (26.1%) (17.9% towards the interatrial septum, 8.2% lateral)
Central	306 (34.9%)

Data are presented as mean (standard deviation), unless otherwise indicated.

AMVL, anterior mitral valve leaflet; MV, mitral valve; PMVL, posterior mitral valve leaflet; TV, tricuspid valve; TVSL, tricuspid valve septal leaflet.

### Tricuspid and mitral valve histological findings in two explanted hearts

Analysis of two explanted whole hearts with ATTR‐CM (hATTR‐CM with the Ser23Asn genetic variant and wtATTR‐CM) was carried out, demonstrating morphological abnormalities in all the different components of the AV apparatus, namely the annulus, leaflets and commissures, chordae tendinae, papillary muscles and the surrounding atrial and ventricular myocardium. These were due not only to the presence of amyloid deposits but also due to remodelling of different components of the valvular apparatus.

**Figure 5:**
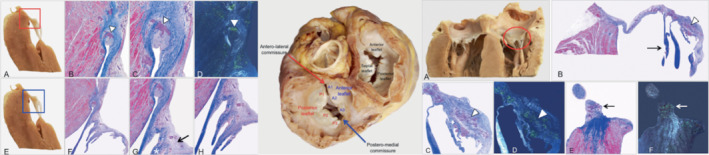
Histological appearances of the mitral and tricuspid valve. Native heart of a 44‐year‐old lady with hereditary transthyretin amyloid cardiomyopathy associated with the Ser23Asn variant. Left panel: Mitral valve posterior leaflet. (*A–D*) Amyloid deposits within the fibrous annulus (arrowheads). (*A*) Macroscopic specimen; (*B*) Azan Mallory (AM) trichrome, original magnification (OM) 25x; (*C*) AM trichome, OM 50x; (*D*) Congo red (CR) staining, OM 50x. (*E–H*) Fibrosis is increased and distributed along the ventricular side (*G*, asterisk); loose connective tissue rich in glycosaminoglycans evident in the atrial side (*G*, arrow). (*E*) Macroscopic specimen; (*F*) scanned slide; (*G*) AM trichrome, OM 25x; (*H*) AM trichrome, OM 25x. Middle panel: Macroscopic view of atrio‐ventricular valves with stiffened annulus, thickened leaflets, loss of normal scallop segmentation. Right panel: (*A*) Macroscopic specimen of the anterior leaflet of the tricuspid valve, which appears swollen and stiffened, due to numerous nodular amyloid deposits (*B–D*) (arrowheads) and fibrous tissue. Tendinous cords are markedly thickened (*B*, arrow). (*E,F*) The apex of papillary muscle indicates fibrosis in blue and multiple amyloid deposits (arrows). (*B*) Scanned slide; (*C*) AM trichrome, OM 25x; (*D*) CR staining, OM 25x; (*E*) AM trichrome, OM 25x; (*F*) CR staining, OM 25x.

**Figure 6:**
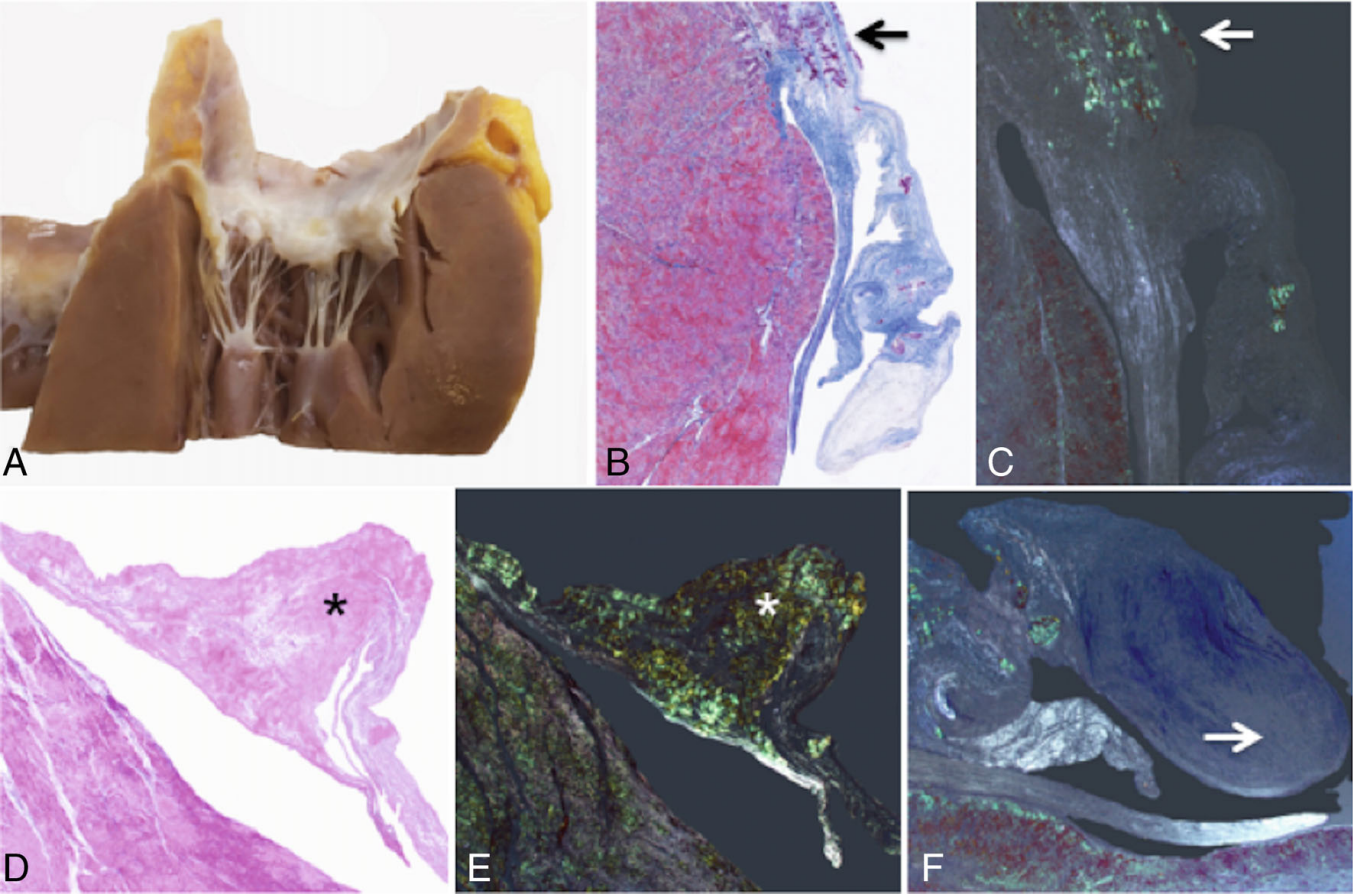
Histological appearance of the mitral valve in wild‐type transthyretin amyloid cardiomyopathy (wtATTR‐CM). Native heart of a 64‐year‐old male with wtATTR‐CM. (*A*) Macroscopic specimen of mitral valve posterior leaflet. (*B–E*) Extensive amyloid deposits in both annulus (*B,C*, arrows) and valve cusp leaflet (*D,E*, asterisks). (*F*) Glycosaminoglycan pooling along the free edge (arrow). (*B*) Scanned slide; (*C*) Congo red staining (CR), original magnification (OM) 25x; (*D*) haematoxylin‐eosin, OM 25x; (*E,F*) CR staining, OM 25x.

At macroscopy (*Figure* *5*, middle panel), both AV leaflets appeared stiff and markedly thickened with loss of normal scallop segmentation. The commissures were enlarged and the annulus was stiffened. The chordae tendinae were thickened and shortened; at their insertion point, the papillary muscle fibrous cap appeared prominent. At histology, the normal layered organization of the leaflets was extensively altered. Thickening of valve leaflets was characterized by increased fibrous tissue in the *fibrosa* layer, pooling of glycosaminoglycans in the spongy layer and alterations to the central core of loose connective tissue, with an associated decrease in plasticity and sliding motion of the layers. Furthermore, there were multiple nodular amyloid deposits, which also extended to the fibrous annulus in various degrees. The surrounding atrial and ventricular myocardium showed extensive interstitial amyloid deposits, which cause stiffening and remodelling of the entire valvular apparatus (see *Figures* *5* and *6* for histological appearances of the mitral and tricuspid valves; and online supplementary *Appendix* *S1* for further detailed histological results).

## Discussion

This is the first study to assess serial echocardiographic parameters and interpret their significance in ATTR‐CM and compare them across three leading TTR genotypes. Patients with V122I‐hATTR‐CM demonstrated the most rapid rate of disease progression and T60A‐hATTR‐CM the slowest. These data also describe for the first time the prognostic implications of changes in a variety of echocardiographic variables at 12‐ and 24‐month follow‐up, identifying worsening mitral and tricuspid regurgitation as the sole parameters of change that predicted ongoing prognosis, with worsening in MR remaining independent after adjusting for known prognostic markers. These novel findings have immediate implications for tracking progression, and potentially regression following treatment, of ATTR‐CM patients in trials and in the clinic.

Our findings indicate evidence of echocardiographic disease progression across all genotypic subgroups of ATTR‐CM at 12‐ and 24‐month follow‐up, comprising worsening parameters of LV systolic function, diastolic function, and right heart structure and function. This is entirely consistent with the inexorable and gradually progressive nature of the clinical condition which is characterized by progressive worsening in the restrictive phenotype with an associated reduction in cavity size, increased wall thickness (leading to a more severe degree of concentric hypertrophy), worsening diastolic function and reduction in deformation and non‐deformation based parameters with a progressive reduction in SV. The three most common genotypic subgroups of ATTR‐CM in the UK (and the USA) are wtATTR‐CM, V122I‐hATTR‐CM, and T60A‐hATTR‐CM. By comparing the differential rate of disease progression between these three genotypes, we demonstrated that patients with V122I‐hATTR‐CM have more rapid decline in biventricular systolic dysfunction, diastolic dysfunction, concentric remodelling as well as in parameters of right heart structure and function compared to the other two genotypic subgroups. Contrastingly, patients with T60A‐hATTR‐CM had the slowest decline in these echocardiographic parameters. Our results support the hypothesis that patients with these three genotypic subtypes are not only diagnosed at different stages in the natural history of their cardiomyopathy but also progress at different rates, even after adjusting for the degree of dysfunction at baseline, supporting the hypothesis of intrinsic differences in disease biology between genotypes, similar to other studies.^4^, ^20^, ^21^

Whilst our results demonstrate that over a 12‐ and 24‐month timepoint, there is a subtle but clear trend towards population level worsening in the main structural and functional parameters, with the observed differences in rate of progression between genotypes, changes in all deformation and non‐deformation based parameters between different timepoints at an individual level were not found to be associated with mortality with the exception of worsening in mitral and tricuspid regurgitation. The reason for the lack of an association between prognosis and structural, diastolic and systolic functional parameters could be related to the differences in the degree of accuracy and precision of different parameter measurements by echocardiography. Accuracy, which is related to systematic bias errors, is extremely important for diagnostic purposes and single timepoint assessments. Precision, which relates to random errors due to noise, refers to the repeatability or consistency of a measurement. Whilst many deformation‐based parameters, such as longitudinal strain, systolic apex to base ratio and relative apical LS have been extensively proven to have good accuracy, with good diagnostic and prognostic capabilities at single timepoint assessment, the precision of these measures seems to be insufficient, especially when changes over time in these parameters are considered (*Table* *1*), and shown to be minimal over a 2‐year period.

Our findings add nuance to the pathophysiological model traditionally associated with ATTR cardiac amyloidosis. Cardiac amyloidosis has been classically characterized as a disease of the myocardium where extracellular amyloid infiltration causes concentric hypertrophy and myocardial stiffening, diastolic and systolic dysfunction, resulting in an upward and leftward shift in the end‐diastolic pressure–volume relationship, with a concomitant decline in forward SV (*Graphical Abstract*). Our findings confirm this model but also emphasize the underlying importance of worsening in mitral and tricuspid regurgitation, as even small changes in the degree of MR and TR, are sufficient to break the haemodynamic equilibrium resulting in a further significant decrease in SV and worse prognosis. It is important to note that it is worsening over time of MR and TR which has a negative impact on prognosis and not the degree of severity *per se*. In patients with ATTR‐CM, worsening in regurgitation of the mitral and tricuspid valve, caused by amyloid infiltration and changes in haemodynamic conditions, by further reducing the low and fixed forward SV, has an impact on patient prognosis. Importantly, worsening in MR remains independent also after adjusting for known predictors, highlighting the important role of this mechanism. In patients with ATTR‐CM, on echocardiography, changes in the mitral and tricuspid valves can range from mild involvement to severe infiltration affecting the entirety of the valve apparatus. These changes include diffuse thickening of the leaflets with reduced mobility of the PMVL that is often shortened or completely hidden, with the entire leaflet being englobed into the adjacent myocardium. The increased thickness and stiffness of papillary muscles and chordae tendinae results in symmetric or asymmetric tethering of the leaflets, causing central or eccentric regurgitation. The non‐dilated but severely infiltrated mitral annulus is associated with upwards displacement of the PMVL, which, in association with the severely impaired ventricular longitudinal function, negatively affects the balance between closing and tethering forces, causing significant changes in the dynamics of the mitral valve apparatus during the cardiac cycle. Of note, 16% of patients with TR had a dilated tricuspid annulus, suggesting a possible contribution of secondary components (MR worsening, pulmonary hypertension and RV to pulmonary circulation uncoupling) towards the mechanism of TR. In the limited histological analysis of the two explanted hearts, macroscopic inspection confirmed the echocardiographic findings. On histology we confirmed that these changes were due to extensive primary amyloid deposition in all the different components of the valvular apparatus with associated remodelling of both the valve and the sub‐valvular apparatus. Of note, there was no evidence of calcification throughout all the different components of the AV valve apparatus.

In this study, we focused primarily on the AV valves, although several studies^22^, ^23^, ^24^ have reported an association between amyloidosis and aortic stenosis in the elderly population. Whilst aortic stenosis should be considered a comorbidity rather than solely a consequence of the amyloid infiltration, the hypothesis that amyloid deposits could contribute to the initiation or acceleration of degenerative aortic stenosis should be explored.

Our findings have immediate implications for clinicians, by providing robust information on the role of echocardiography in tracking disease progression in this population for the first time. However, they also raise the possibility of shifting therapeutic interventions from the myocardium to the myocardium and the valves. The framework for clinical decision‐making in valvular pathology has drastically changed over the last few years. Mitral and tricuspid valve regurgitation in ATTR‐CM is determined by multiple complex interactions, where valvular, sub‐valvular, haemodynamic and ventricular mechanisms all contribute to the degree of MR and TR, but most importantly have a direct impact on patient prognosis. The crucial question for the future will not only be to fully characterize and quantify the determinants of MR in patients with ATTR‐CM, but to also determine whether an intervention directed at the mitral valve will be capable of changing the clinical course of the disease. Future studies will be needed to address this important clinical question.

### Study limitations

We used a nuanced grading scheme (from grade 0 to 5) for assessing both MR and TR severity adapting the current recommended multiparametric approach. This was chosen as in patients with chronic low flow status even in the presence of diffuse structural valvular abnormalities, the degree of regurgitant volumes should be revised to reflect the low flow condition. A five degree scheme was therefore chosen as it was thought to better reflect the regurgitation in this population with restrictive physiology. However, in the absence of a universal definition, the grading scheme selected could have limitations, especially because one grade (moderate) was subdivided into three grades and because this classification can be more difficult to reproduce in clinical practice.^17^ Wherever a quantitative evaluation was not available a combination of visual estimation and/or semiquantitative parameters were selected by operators. The SV was measured based on estimation of LV volumes, therefore does not account for valvular regurgitation, leading to overestimation of SV in the presence of significant MR. Doppler‐derived SV which could have overcome this limitation was not available for all patients in the present population. We performed strain analysis only in a four‐chamber view as good quality imaging without segment drop out is more challenging in two‐ or three‐chamber apical views compared to four‐chamber views.^25^ This approach was preferred to the global LS to minimize excluded patients and has been previously validated in a large study of over 1000 patients with ATTR‐CM.^5^ Furthermore, good correlation between LS in the three apical views and four‐chamber LS has been demonstrated.^25^ We performed strain analysis using a single vendor software (Echo PAC software, GE), and whilst we acknowledge that inter‐vendor variability has been reported, it has been demonstrated that mean LV global LS values obtained from the software used in this study are not dissimilar from other mainstream providers.^26^ This study has a selection bias, i.e. only patients who are fit to come back to the centre for a repeated follow‐up are assessed in this study and we have now acknowledged this limitation. However, this bias would be the same for any study assessing changes over time on any imaging modality, blood test (NT‐proBNP for example) or any other medical assessment, as the study cohort will focus only on patients able to come back to the centre. Finally, the histological analsis has only been perfomed in two explanted hearts and cannot be considered a systematic assessment.

## Conclusion

Serial echocardiography in patients with ATTR‐CM shows progressive remodelling with worsening parameters of systolic and diastolic function, as well as deformation‐based parameters, with V122I‐hATTR‐CM demonstrating the most rapid deterioration. Worsening in MR and TR are the only echocardiographic change parameters independently associated with mortality. Further studies will be needed to understand if valvular interventions may have a role in this population.

## Supporting information


**Appendix S1**. Supporting information.
