# Supplementary material for: Progression of echocardiographic parameters and prognosis in transthyretin cardiac amyloidosis
Source: Eur J Heart Fail. 2022 Jul 27;24(9):1700–12. doi: 10.1002/ejhf.2606 (PMC10108569; doi:10.1002/ejhf.2606)
Supplement: Supplementary file 1 — Appendix S1. Supporting information. [file EJHF-24-1700-s001.docx]

**Supplemental Appendix**

[Supplemental methods: Echocardiography 2](#_Toc102508719)

[Supplemental Figure 1. Consort diagram 5](#_Toc102508720)

[Supplemental Table 1. Linear regression analysis comparing patients with V122I vs wtATTR-CM 6](#_Toc102508721)

[Supplemental Table 2. Linear regression analysis comparing patients with T60A vs wtATTR-CM 9](#_Toc102508722)

[Supplemental Results: Valve Progression 13](#_Toc102508723)

[Supplemental Results: Tricuspid and mitral valve histological findings in two explanted hearts 15](#_Toc102508724)

# Supplemental methods: Echocardiography

Echocardiographic evaluation was performed using a GE Vivid E9 ultrasound machine equipped with a 5S probe and measurements performed offline using EchoPAC software (Version 202). At least 3 consecutive beats were recorded for each view, and images were stored for off-line analysis. LV chamber quantification was assessed following the latest American Society of Echocardiography/European Association of Cardiovascular Imaging Guideline (1): LV mass was calculated using Devereux’s formula, relative wall thickness (RWT) was calculated as 2*posterior wall thickness in diastole/Left ventricular end diastolic diameter (2*PWTd/LVEDD), stroke volume (SV) was calculated as SV was calculated as: end diastolic volume (EDV) − end systolic volume (ESV), myocardial volume (MVol) as LV mass/1.05g/ml, and myocardial contraction fraction (MCF) as stroke volume (SV) /MVol, as previously reported. (2) LA dimensions were reported in the parasternal long-axis view and absolute 4-chambers area. Right atrial area (RAA) was measured in the 4-chamber view. LV ejection fraction (EF) was calculated with the biplane Simpson’s method from volumes acquired in both the 4-chamber and the 2-chamber views. Lateral mitral annular plane systolic excursion (MAPSE) and tricuspid annular plane systolic excursion (TAPSE) were assessed with M-mode in the 4-chamber view. LV early (E wave), late (A wave) diastolic filling, its ratio (E/A) were evaluated with pulsed Doppler in the 4-chamber view. Lateral and septal mitral annulus velocity (e’ wave) was assessed with tissue Doppler in the 4-chamber view; the ratio between the LV early diastolic filling wave and lateral mitral annulus velocity (E/e’) was calculated.(3) Pulmonary artery systolic pressure (PASP) was estimated based on the peak tricuspid regurgitation (TR) velocity, as described by the simplified Bernoulli equation, taking into account right atrial pressure , as estimated based on the diameter and respiratory variation in diameter of the inferior vena cava. (1, 4) The TAPSE/PASP ratio was calculated, as a non-invasive index of right ventricle to pulmonary circulation coupling.(5) Digitally acquired clips were considered suitable for offline 2D Speckle Strain Imaging analysis if at least three cardiac cycles were available, with high frame rates (70 to 100 frame/s) and without dropout of more than one LV segment or significant foreshortening of the ventricle. The endocardial border was traced at the end-diastolic frame in the apical view. End-diastole was defined by the QRS complex or by the frame just before mitral valve closure. The software tracked speckles along the endocardial and epicardial borders throughout the cardiac cycle, and the width of the region of interest was adjusted to fit the entire myocardium. All strain and strain-derived variables were measured in the apical 4-chamber view. Peak longitudinal strain (LS) was computed automatically, generating regional data from 6 segments (basal, mid, apical interventricular septum and basal, mid, apical lateral wall), to calculate an average value. Strain-derived variables were acquired and calculated according to previous studies: septal longitudinal systolic apex to base (SAB) ratio (6) and relative apical longitudinal strain (RALS) as the average 4-chamber apical segments peak longitudinal strain/average basal and mid 4-chamber peak longitudinal strain .(7) Valvular assessment was performed using an integrated approach as per current guidelines. The evaluation of TR and mitral regurgitation (MR) involved an integrated approach using qualitative characteristics (valve morphology and colour flow regurgitant jet), and semi-quantitative and quantitative measures (vena contracta, effective regurgitant orifice area, regurgitant volume).(8) (9) LA speckle tracking analysis was performed according to current consensus criteria.(10) Briefly, non-foreshortened 2D apical 4-chamber view was used to define a 3 mm-thickened wall region of interest (ROI) along the LA. The LA contour was extrapolated excluding pulmonary veins and LA appendage. Quality control check was performed to reject cases with significant (>1/3 of LA contour) drop out of atrial wall. Zero-baseline was defined as ventricular end-diastole, using the R-R cycle for analysis. LA myocardial deformation was assessed as global longitudinal strain obtained with the endocardial curve of the ROI. Reservoir, contraction and conduit phase were studied respectively as: LA strain (LAS) reservoir= peak value at the onset of LV filling, LAS contraction= peak value at the onset of atrial contraction (for subjects in sinus rhythm).

# Supplemental Figure 1. Consort diagram


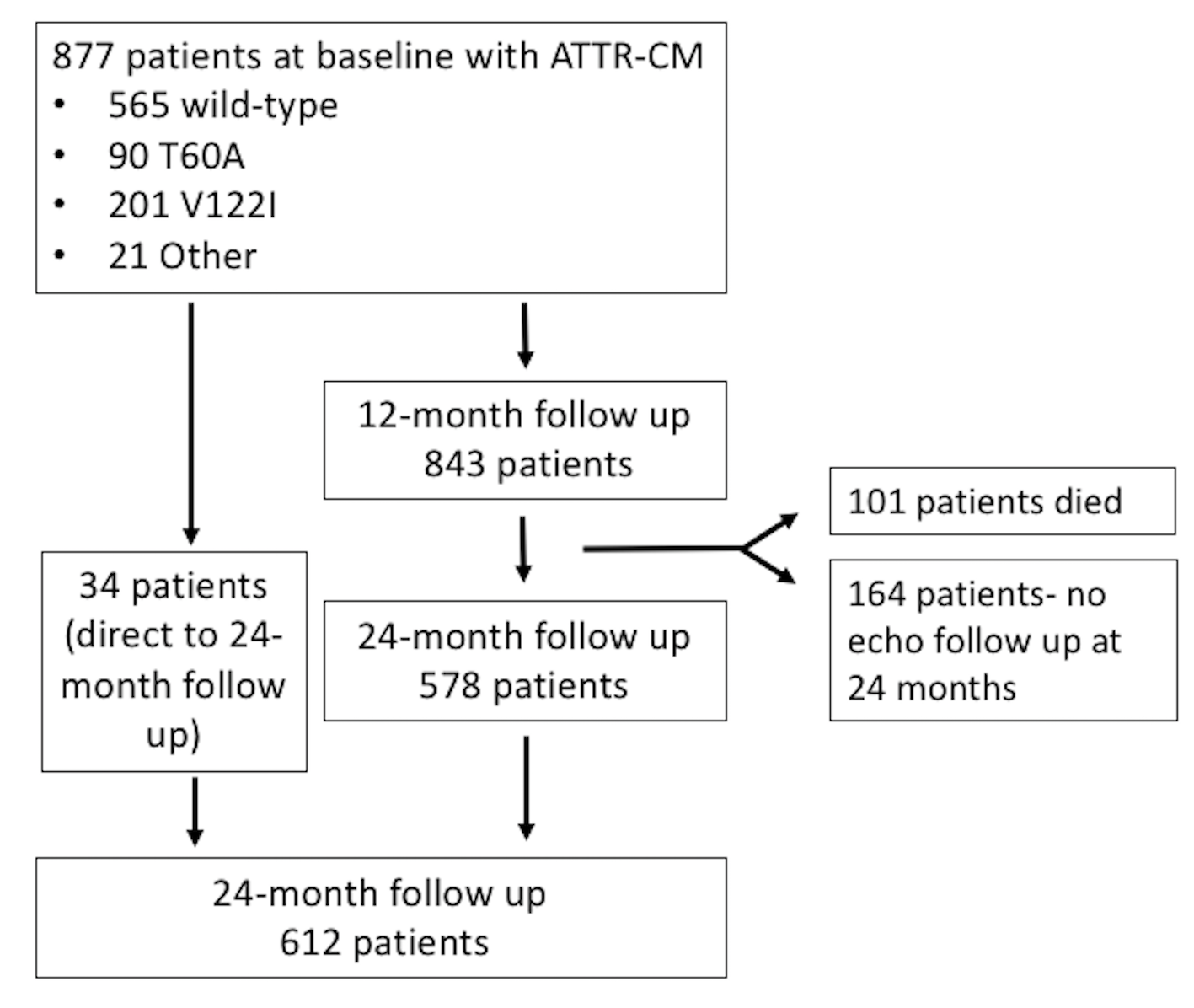


**Figure legend.** Consort diagram representing patients at baseline,12-and 24-month assessment.

# Supplemental Table 1. Linear regression analysis comparing patients with V122I vs wtATTR-CM

|  | **Baseline to 1 year- V122I Versus Wild-type** | | | | | **Baseline to 2 year- V122I Versus Wild-type** | | | | |
| --- | --- | --- | --- | --- | --- | --- | --- | --- | --- | --- |
| **Echocardiographic Variable** | **Regression Coefficient** | **t** | **p-value** | **Lower**  **CI** | **Upper CI** | **Regression Coefficient** | **t** | **p-value** | **Lower CI** | **Upper CI** |
| **IVSd(mm)** | -0.09 | -1.02 | 0.308 | -0.26 | 0.08 | 0.01 | 0.08 | 0.940 | -0.26 | 0.28 |
| **IVSd Index(mm/m^2^)** | 0.02 | 0.32 | 0.750 | -0.08 | 0.11 | 0.09 | 1.20 | 0.230 | -0.06 | 0.24 |
| **LVEDD(mm)** | -0.86 | -2.57 | **0.010** | -1.53 | -0.20 | -1.02 | -2.31 | **0.021** | -1.88 | -0.15 |
| **LVEDD index**  **(mm/m^2^)** | -0.15 | -0.87 | 0.384 | -0.49 | 0.19 | -0.27 | -1.23 | 0.220 | -0.71 | 0.16 |
| **PWTd(mm)** | 0.06 | 0.55 | 0.586 | -0.17 | 0.29 | 0.10 | 0.58 | 0.565 | -0.23 | 0.42 |
| **PWTd Index**  **(mm/m^2^)** | 0.14 | 2.19 | **0.029** | 0.01 | 0.27 | 0.17 | 1.86 | 0.063 | -0.01 | 0.34 |
| **MWT(mm)** | -0.03 | -0.33 | 0.745 | -0.19 | 0.14 | 0.04 | 0.34 | 0.736 | -0.21 | 0.30 |
| **MWT Index**  **(mm/m^2^)** | 0.05 | 1.08 | 0.281 | -0.04 | 0.14 | 0.10 | 1.45 | 0.147 | -0.04 | 0.24 |
| **RWT** | 0.02 | 1.92 | 0.056 | 0.00 | 0.04 | 0.03 | 2.15 | **0.032** | 0.00 | 0.05 |
| **LVESD(mm)** | -0.20 | -0.50 | 0.618 | -0.96 | 0.57 | -0.98 | -2.12 | **0.034** | -1.90 | -0.07 |
| **LVESD Index**  **(mm/m^2^)** | 0.21 | 1.03 | 0.303 | -0.19 | 0.61 | -0.23 | -0.94 | 0.348 | -0.71 | 0.25 |
| **LVEDV(mL)** | -9.34 | -5.07 | **0.000** | -12.96 | -5.72 | -8.34 | -3.82 | **0.000** | -12.63 | -4.05 |
| **LVEDV Index**  **(mL/m^2^)** | -4.41 | -4.67 | **0.000** | -6.26 | -2.56 | -3.82 | -3.41 | **0.001** | -6.03 | -1.62 |
| **LVESV(mL)** | -2.18 | -1.90 | 0.058 | -4.44 | 0.07 | -3.26 | -2.27 | **0.024** | -6.09 | -0.43 |
| **LVESV Index**  **(mL/m^2^)** | -0.82 | -1.39 | 0.165 | -1.99 | 0.34 | -1.27 | -1.72 | 0.086 | -2.73 | 0.18 |
| **SV(mL)** | -6.56 | -6.17 | **0.000** | -8.65 | -4.47 | -6.34 | -5.20 | **0.000** | -8.73 | -3.94 |
| **SV Index**  **(mL/m^2^)** | -3.30 | -6.11 | **0.000** | -4.35 | -2.24 | -3.12 | -5.01 | **0.000** | -4.34 | -1.90 |
| **EF(%)** | -2.05 | -2.67 | **0.008** | -3.55 | -0.54 | -2.10 | -2.31 | **0.021** | -3.89 | -0.31 |
| **LAD(mm)** | -0.86 | -2.09 | **0.037** | -1.66 | -0.05 | -2.00 | -3.81 | **0.000** | -3.03 | -0.97 |
| **LAA 4ch(cm^2^)** | -1.28 | -3.48 | **0.001** | -2.00 | -0.56 | -1.45 | -3.13 | **0.002** | -2.36 | -0.54 |
| **LAA 4ch Index**  **(cm^2^/m^2^)** | -0.35 | -1.81 | 0.070 | -0.74 | 0.03 | -0.31 | -1.26 | 0.208 | -0.78 | 0.17 |
| **RAA 4ch(cm^2^)** | 0.33 | 0.86 | 0.388 | -0.43 | 1.09 | -0.05 | -0.09 | 0.925 | -1.04 | 0.95 |
| **RAA 4ch index**  **(cm^2^/m^2^)** | 0.37 | 1.82 | 0.068 | -0.03 | 0.77 | 0.16 | 0.60 | 0.549 | -0.37 | 0.69 |
| **LVM(g)** | -7.17 | -1.82 | 0.069 | -14.89 | 0.55 | -9.22 | -1.62 | 0.105 | -20.36 | 1.93 |
| **LVM Index(g/m^2^)** | -2.22 | -1.10 | 0.271 | -6.16 | 1.73 | -3.26 | -1.13 | 0.257 | -8.91 | 2.39 |
| **MCF(%)** | -0.01 | -2.89 | **0.004** | -0.02 | 0.00 | -0.01 | -1.44 | 0.150 | -0.01 | 0.00 |
| **DT(ms)** | -13.42 | -2.94 | **0.003** | -22.40 | -4.44 | -15.23 | -2.89 | **0.004** | -25.57 | -4.89 |
| **E/A Ratio** | 0.04 | 0.38 | 0.708 | -0.16 | 0.23 | 0.29 | 2.35 | **0.020** | 0.05 | 0.54 |
| **E' Lateral(cm/s)** | -0.31 | -2.20 | **0.028** | -0.59 | -0.03 | -0.56 | -2.87 | **0.004** | -0.95 | -0.18 |
| **E' Septal(cm/s)** | -0.36 | -3.22 | **0.001** | -0.58 | -0.14 | -0.39 | -2.64 | **0.009** | -0.68 | -0.10 |
| **E/e' lateral** | 0.03 | 0.06 | 0.955 | -0.86 | 0.91 | 0.76 | 1.18 | 0.240 | -0.51 | 2.03 |
| **E/e' average** | 0.12 | 0.28 | 0.778 | -0.70 | 0.93 | 0.34 | 0.57 | 0.569 | -0.84 | 1.52 |
| **MAPSE(mm)** | -0.59 | -2.92 | **0.004** | -0.98 | -0.19 | -0.91 | -3.84 | **0.000** | -1.38 | -0.45 |
| **TAPSE(mm)** | -0.46 | -1.43 | 0.153 | -1.10 | 0.17 | -0.72 | -1.74 | 0.082 | -1.53 | 0.09 |
| **S' tricuspid(cm/s)** | -0.41 | -1.78 | 0.075 | -0.86 | 0.04 | -0.84 | -2.87 | **0.004** | -1.41 | -0.26 |
| **TR Gradient**  **(mmHg)** | 0.17 | 0.19 | 0.847 | -1.55 | 1.89 | 1.98 | 1.66 | 0.098 | -0.36 | 4.32 |
| **PASP (mmHg)** | 0.78 | 0.77 | 0.443 | -1.22 | 2.79 | 1.91 | 1.50 | 0.135 | -0.60 | 4.43 |
| **LV LS (%)** | 0.64 | 2.45 | **0.014** | 0.13 | 1.15 | 0.88 | 2.69 | **0.007** | 0.24 | 1.53 |
| **SABr** | -0.61 | -1.09 | 0.276 | -1.71 | 0.49 | -0.51 | -0.63 | 0.531 | -2.09 | 1.08 |
| **RALS** | 0.28 | 1.31 | 0.189 | -0.14 | 0.69 | -0.13 | -0.51 | 0.610 | -0.63 | 0.37 |
| **TAPSE/PASP** | -0.02 | -1.02 | 0.310 | -0.05 | 0.02 | -0.02 | -0.90 | 0.369 | -0.05 | 0.02 |
| **LA Strain Res** | -0.01 | -0.02 | 0.986 | -1.44 | 1.42 | -1.29 | -1.58 | 0.115 | -2.89 | 0.31 |
| **LA Strain Contract** | 1.09 | 1.32 | 0.188 | -0.54 | 2.72 | -1.24 | -1.20 | 0.232 | -3.27 | 0.80 |
| **RV LS (%)** | 0.39 | 1.33 | 0.184 | -0.19 | 0.97 | 0.77 | 1.86 | 0.064 | -0.05 | 1.59 |

**Table legend.** Linear regression analysis comparing patients with V122I vs wtATTR-CM. Statistical significance, shown in bold, is represented by p-values < 0.05. Data are presented as regression co-efficient, p-value, confidence interval. The regression co-efficient signifies a reduction or increase in a given parameter in genotype V122I when compared to wild-type at the given timepoints.

# Supplemental Table 2. Linear regression analysis comparing patients with T60A vs wtATTR-CM

|  | **Baseline to 1 year- T60A Versus Wild-type** | | | | | **Baseline to 2 year- T60A Versus Wild-type** | | | | |
| --- | --- | --- | --- | --- | --- | --- | --- | --- | --- | --- |
| **Echocardiographic**  **Variable** | **Regression Coefficient** | **t** | **p-value** | **Lower CI** | **Upper CI** | **Regression Coefficient** | **t** | **p-value** | **Lower CI** | **Upper CI** |
| **IVSd(mm)** | -0.180 | -1.338 | 0.181 | -0.444 | 0.084 | 0.016 | 0.082 | 0.935 | -0.363 | 0.395 |
| **IVSd Index(mm/m^2^)** | 0.039 | 0.536 | 0.592 | -0.103 | 0.18 | 0.202 | 1.943 | 0.053 | -0.002 | 0.406 |
| **LVEDD(mm)** | -0.579 | -1.153 | 0.249 | -1.566 | 0.407 | -0.681 | -1.134 | 0.257 | -1.861 | 0.498 |
| **LVEDD index**  **(mm/m^2^)** | 0.336 | 1.276 | 0.202 | -0.181 | 0.853 | 0.223 | 0.712 | 0.476 | -0.392 | 0.838 |
| **PWTd(mm)** | -0.243 | -1.362 | 0.174 | -0.593 | 0.107 | -0.157 | -0.679 | 0.498 | -0.61 | 0.297 |
| **PWTd Index**  **(mm/m^2^)** | 0.114 | 1.157 | 0.248 | -0.079 | 0.307 | 0.183 | 1.452 | 0.147 | -0.065 | 0.431 |
| **MWT(mm)** | -0.2 | -1.598 | 0.110 | -0.446 | 0.046 | -0.06 | -0.327 | 0.744 | -0.417 | 0.298 |
| **MWT Index**  **(mm/m^2^)** | 0.031 | 0.449 | 0.654 | -0.103 | 0.165 | 0.156 | 1.584 | 0.114 | -0.037 | 0.349 |
| **RWT** | -0.002 | -0.168 | 0.867 | -0.029 | 0.025 | 0.001 | 0.058 | 0.954 | -0.034 | 0.036 |
| **LVESD(mm)** | -0.168 | -0.282 | 0.778 | -1.335 | 0.999 | -1.579 | -2.425 | **0.016** | -2.857 | -0.3 |
| **LVESD Index**  **(mm/m^2^)** | 0.494 | 1.584 | 0.114 | -0.118 | 1.106 | -0.318 | -0.924 | 0.356 | -0.993 | 0.357 |
| **LVEDV(mL)** | -2.796 | -0.982 | 0.326 | -8.385 | 2.793 | -8.701 | -2.843 | **0.005** | -14.712 | -2.691 |
| **LVEDV Index**  **(mL/m^2^)** | 0.083 | 0.057 | 0.955 | -2.787 | 2.954 | -2.822 | -1.784 | 0.075 | -5.93 | 0.285 |
| **LVESV(mL)** | -2.146 | -1.188 | 0.235 | -5.69 | 1.399 | -7.792 | -3.797 | **0.000** | -11.822 | -3.762 |
| **LVESV Index**  **(mL/m^2^)** | -0.597 | -0.644 | 0.520 | -2.416 | 1.222 | -3.413 | -3.238 | **0.001** | -5.483 | -1.343 |
| **SV(mL)** | -1.056 | -0.661 | 0.509 | -4.191 | 2.079 | -0.826 | -0.495 | 0.621 | -4.101 | 2.45 |
| **SV Index**  **(mL/m^2^)** | 0.407 | 0.496 | 0.62 | -1.203 | 2.016 | 0.840 | 0.972 | 0.331 | -0.857 | 2.538 |
| **EF(%)** | -0.017 | -0.015 | 0.988 | -2.29 | 2.256 | 4.062 | 3.18 | **0.002** | 1.553 | 6.57 |
| **LAD(mm)** | -0.847 | -1.355 | 0.176 | -2.074 | 0.38 | -0.918 | -1.214 | 0.225 | -2.403 | 0.567 |
| **LAA 4ch(cm^2^)** | -0.583 | -1.022 | 0.307 | -1.703 | 0.537 | -1.255 | -1.872 | 0.062 | -2.572 | 0.062 |
| **LAA 4ch Index**  **(cm^2^/m^2^)** | 0.305 | 1.02 | 0.308 | -0.282 | 0.893 | -0.027 | -0.079 | 0.937 | -0.705 | 0.65 |
| **RAA 4ch(cm)** | -0.076 | -0.125 | 0.901 | -1.267 | 1.116 | -1.452 | -1.950 | 0.052 | -2.914 | 0.01 |
| **RAA 4ch index**  **(cm^2^/m^2^)** | 0.354 | 1.117 | 0.265 | -0.268 | 0.976 | -0.484 | -1.248 | 0.213 | -1.247 | 0.278 |
| **LVM(g)** | -10.019 | -1.678 | 0.094 | -21.74 | 1.701 | -10.081 | -1.271 | 0.204 | -25.66 | 5.498 |
| **LVM Index(g/m^2^)** | -1.656 | -0.542 | 0.588 | -7.653 | 4.34 | -0.736 | -0.182 | 0.855 | -8.655 | 7.183 |
| **MCF(%)** | -0.001 | -0.157 | 0.875 | -0.011 | 0.009 | 0.005 | 0.829 | 0.407 | -0.007 | 0.016 |
| **DT(ms)** | -3.622 | -0.528 | 0.597 | -17.081 | 9.836 | 17.379 | 2.261 | **0.024** | 2.28 | 32.478 |
| **E/A Ratio** | -0.158 | -1.091 | 0.276 | -0.443 | 0.127 | -0.558 | -3.092 | **0.002** | -0.914 | -0.203 |
| **E' Lateral(cm/s)** | -0.001 | -0.005 | 0.996 | -0.428 | 0.426 | -0.417 | -1.497 | 0.135 | -0.965 | 0.13 |
| **E' Septal(cm/s)** | -0.099 | -0.567 | 0.571 | -0.444 | 0.245 | -0.089 | -0.408 | 0.684 | -0.52 | 0.341 |
| **E/e' lateral** | 1.679 | 2.407 | **0.016** | 0.31 | 3.047 | 2.272 | 2.379 | **0.018** | 0.396 | 4.149 |
| **E/e' average** | 1.707 | 2.594 | **0.010** | 0.415 | 2.999 | 1.97 | 2.133 | **0.033** | 0.156 | 3.784 |
| **MAPSE(mm)** | -0.14 | -0.477 | 0.633 | -0.715 | 0.435 | 0.275 | 0.806 | 0.420 | -0.395 | 0.945 |
| **TAPSE(mm)** | -0.0920 | -0.192 | 0.848 | -1.035 | 0.851 | 1.492 | 2.520 | **0.012** | 0.329 | 2.654 |
| **S' tricuspid(cm/s)** | -0.156 | -0.44 | 0.660 | -0.85 | 0.539 | 0.736 | 1.788 | 0.074 | -0.073 | 1.545 |
| **TR Gradient**  **(mmHg)** | 0.617 | 0.433 | 0.665 | -2.184 | 3.419 | -1.409 | -0.813 | 0.416 | -4.814 | 1.996 |
| **PASP(mmHg)** | -0.284 | -0.17 | 0.865 | -3.566 | 2.997 | -2.378 | -1.247 | 0.213 | -6.127 | 1.371 |
| **LV LS(%)** | -0.073 | -0.181 | 0.856 | -0.866 | 0.72 | -0.181 | -0.387 | 0.699 | -1.101 | 0.738 |
| **SAB** | 0.042 | 0.048 | 0.962 | -1.674 | 1.757 | -1.285 | -1.098 | 0.273 | -3.584 | 1.013 |
| **RELAPSI** | 0.444 | 1.346 | 0.179 | -0.204 | 1.092 | -0.1 | -0.271 | 0.786 | -0.823 | 0.624 |
| **TAPSE/PASP** | -0.003 | -0.122 | 0.903 | -0.06 | 0.053 | 0.05 | 1.78 | 0.076 | -0.005 | 0.105 |
| **LA Strain Res** | 1.699 | 1.462 | 0.144 | -0.584 | 3.981 | 3.057 | 2.552 | **0.011** | 0.702 | 5.412 |
| **LA Strain Contract** | 3.433 | 2.375 | **0.019** | 0.582 | 6.284 | 4.964 | 2.775 | **0.007** | 1.413 | 8.515 |
| **RV LS(%)** | -0.809 | -1.755 | 0.080 | -1.714 | 0.097 | -1.55 | -2.629 | **0.009** | -2.709 | -0.391 |

**Table legend.** Linear regression analysis comparing patients with T60A vs wtATTR-CM. Statistical significance, shown in bold, is represented by p-values < 0.05. Data are presented as regression co-efficient, p-value, confidence interval. The regression co-efficient signifies a reduction or increase in a given parameter in genotype T60A when compared to wild-type at the given timepoints.

# Supplemental Results: Valve Progression

**Mitral Regurgitation-12 months**

At 12 months, 215(25.5%) patients with MR had progressed by a grade of at least ‘1’, of whom at baseline,103(47.9%) had no MR, 81(37.7%) had ‘mild’ MR, 23(10.7%) had ‘mild-to-moderate’ MR, 6(2.8%) had ‘moderate’ MR and 2(0.9%) had ‘moderate-to-severe’ MR. At 12 months,70 (8.3%) patients with MR had progressed by a grade of at least ‘2’,of whom at baseline,32 (45.7%) had no MR, 31 (44.3%) had ‘mild’ MR, 5 (7.1%) had ‘mild to moderate’ MR and 2 (2.9%) had ‘moderate’ MR.

**Mitral Regurgitation-24 months**

At 24 months, 204 (33.3%) patients with MR progressed from baseline assessment by ‘at least’ a grade of 1 of whom at baseline,120 (58.8%) had no MR, 58 (28.4%) had ‘mild’ MR, 22 (10.8%) had ‘mild-to-moderate’ MR, 4 (2.0%) had ‘moderate’ MR. At 24 months, 69 (11.3%) patients with MR progressed from baseline assessment by ‘at least’ a grade of 2 of whom at baseline,37 (53.6%) had no MR, 27 (39.1%) had ‘mild’ MR, 4 (5.8%) had ‘mild-to-moderate’ MR, 1 (1.5%) had ‘moderate’ MR.

**Tricuspid Regurgitation 12 months**

At 12 months, 214 (25.4%) patients with TR had progressed by at least grade of ‘1’ or above, of whom at baseline, 98 (45.8%) had no TR, 77 (36%) had ‘mild’ TR, 27 (12.6%) had ‘mild to moderate’ TR, 8 (3.7%) had ‘moderate’ TR and 4 (1.9%) had ‘moderate-to-severe’ TR at baseline. At 12 months, 91(10.8%) patients with TR had progressed by a grade of at least ‘2’, of whom at baseline, 35 (38.5%) had no TR, 41 (45%) had ‘mild’ TR 10 (11%) had ‘mild to moderate’ TR, 5 (5.5%) had ‘moderate’ TR.

**Tricuspid Regurgitation 24 months**

At 24 months, 227 (37.1%) patients with TR progressed from baseline assessment by ‘at least’ a grade of 1 of whom at baseline,112 (49.3%) had no TR, 79 (34.8%) had ‘mild’ TR, 21 (9.3%) had ‘mild-to-moderate’ MR, 13 (5.7%) had ‘moderate’ TR and 2 (0.9%) had ‘moderate-to-severe’ TR. At 24 months, 77 (12.6%) of patients with TR progressed from baseline assessment by ‘at least’ a grade of 2 of whom at baseline, 29 (37.7%) had no TR, 37 (48.1%) had ‘mild’ TR, 5 (6.5%) had ‘mild-to-moderate’ TR, 6 (7.8%) had ‘moderate’ TR.

# Supplemental Results: Tricuspid and mitral valve histological findings in two explanted hearts

Analysis of two explanted whole hearts with ATTR-CM(hATTR-CM with the Ser23Asn genetic variant and wtATTR-CM) was carried out,demonstrating morphological abnormalities in all the different components of the AV apparatus, namely the annulus, leaflets and commissures,chordae tendinae,papillary muscles and the surrounding atrial and ventricular myocardium.These were due not only to the presence of amyloid deposits but also due to remodelling of different components of the valvular apparatus.

At macroscopy(figure 5, middle panel, main manuscript), both AV leaflets appeared stiff and markedly thickened with loss of normal scallop segmentation.The commissures were enlarged and the annulus was stiffened.The chordae tendinae were thickened and shortened; at their insertion point, the papillary muscle fibrous cap appeared prominent.At histology, the normal layered organization of the leaflets was extensively altered.Thickening of valve leaflets was characterized by increased fibrous tissue in the fibrosa layer, pooling of glycosaminoglycans in the spongy layer and alterations to the central core of loose connective tissue,with an associated decrease in plasticity and sliding motion of the layers.Furthermore, there were multiple nodular amyloid deposits, which also extended to the fibrous annulus in various degrees.The surrounding atrial and ventricular myocardium showed extensive interstitial amyloid deposits, which cause stiffening and remodelling of the entire valvular apparatus.The mitral valve posterior leaflet(Figure 5, left panel, main manuscript) appeared thickened due to fibrosis of the ventricular and fibrosa layers, with associated extension of the spongy layer towards the atrial layer.The leaflet showed extensive fibrosis in its proximal segment and nodular pooling of glycosaminoglycans along the free edge.Amyloid deposits were scattered throughout the leaflet, and also involved the annulus with Congo red stain confirming the typical green birefringence of amyloid deposits (Figure 5 and 6, main manuscript).The chordae tendinae appeared thickened due to increased compact fibrous tissue with loss of both normal collagen fibrils and the peripheral loosely arranged collagen.The anterior leaflet of the tricuspid valve(Figure 5, right panel, main manuscript) had numerous nodular amyloid deposits in the context of extensive fibrous tissue.The papillary muscle showed thickening of the fibrotic component at the apex and multiple amyloid deposits.There was no evidence of calcification in all the different components of the AV valve apparatus.

**References**

1. Lang RM, Badano LP, Mor-Avi V, Afilalo J, Armstrong A, Ernande L, et al. Recommendations for cardiac chamber quantification by echocardiography in adults: an update from the American Society of Echocardiography and the European Association of Cardiovascular Imaging. Eur Heart J Cardiovasc Imaging. 2015;16(3):233-70.

2. Tendler A, Helmke S, Teruya S, Alvarez J, Maurer MS. The myocardial contraction fraction is superior to ejection fraction in predicting survival in patients with AL cardiac amyloidosis. Amyloid. 2015;22(1):61-6.

3. Nagueh SF, Smiseth OA, Appleton CP, Byrd BF, 3rd, Dokainish H, Edvardsen T, et al. Recommendations for the Evaluation of Left Ventricular Diastolic Function by Echocardiography: An Update from the American Society of Echocardiography and the European Association of Cardiovascular Imaging. Eur Heart J Cardiovasc Imaging. 2016;17(12):1321-60.

4. Galie N, Humbert M, Vachiery JL, Gibbs S, Lang I, Torbicki A, et al. 2015 ESC/ERS Guidelines for the diagnosis and treatment of pulmonary hypertension: The Joint Task Force for the Diagnosis and Treatment of Pulmonary Hypertension of the European Society of Cardiology (ESC) and the European Respiratory Society (ERS): Endorsed by: Association for European Paediatric and Congenital Cardiology (AEPC), International Society for Heart and Lung Transplantation (ISHLT). Eur Heart J. 2016;37(1):67-119.

5. Guazzi M, Dixon D, Labate V, Beussink-Nelson L, Bandera F, Cuttica MJ, et al. RV Contractile Function and its Coupling to Pulmonary Circulation in Heart Failure With Preserved Ejection Fraction: Stratification of Clinical Phenotypes and Outcomes. JACC Cardiovasc Imaging. 2017;10(10 Pt B):1211-21.

6. Liu D, Hu K, Niemann M, Herrmann S, Cikes M, Stork S, et al. Effect of combined systolic and diastolic functional parameter assessment for differentiation of cardiac amyloidosis from other causes of concentric left ventricular hypertrophy. Circ Cardiovasc Imaging. 2013;6(6):1066-72.

7. Phelan D, Collier P, Thavendiranathan P, Popovic ZB, Hanna M, Plana JC, et al. Relative apical sparing of longitudinal strain using two-dimensional speckle-tracking echocardiography is both sensitive and specific for the diagnosis of cardiac amyloidosis. Heart. 2012;98(19):1442-8.

8. Zoghbi WA, Adams D, Bonow RO, Enriquez-Sarano M, Foster E, Grayburn PA, et al. Recommendations for Noninvasive Evaluation of Native Valvular Regurgitation: A Report from the American Society of Echocardiography Developed in Collaboration with the Society for Cardiovascular Magnetic Resonance. J Am Soc Echocardiogr. 2017;30(4):303-71.

9. Lancellotti P, Moura L, Pierard LA, Agricola E, Popescu BA, Tribouilloy C, et al. European Association of Echocardiography recommendations for the assessment of valvular regurgitation. Part 2: mitral and tricuspid regurgitation (native valve disease). Eur J Echocardiogr. 2010;11(4):307-32.

10. Badano LP, Kolias TJ, Muraru D, Abraham TP, Aurigemma G, Edvardsen T, et al. Standardization of left atrial, right ventricular, and right atrial deformation imaging using two-dimensional speckle tracking echocardiography: a consensus document of the EACVI/ASE/Industry Task Force to standardize deformation imaging. Eur Heart J Cardiovasc Imaging. 2018;19(6):591-600.
